# Immediate application of low-intensity electrical noise reduced responses to visual perturbations during walking in individuals with cerebral palsy

**DOI:** 10.1186/s12984-023-01299-1

**Published:** 2024-01-28

**Authors:** Ashwini Sansare, Maelyn Arcodia, Samuel C. K. Lee, John Jeka, Hendrik Reimann

**Affiliations:** 1 Department of Physical Therapy, University of Delaware, Newark, DE USA; 2 Department of Kinesiology and Applied Physiology, University of Delaware, Newark, DE USA

**Keywords:** Stochastic resonance, Sensorimotor integration, Locomotor control, Balance, Virtual reality, Visual reliance

## Introduction

Cerebral palsy (CP) is a neurological disorder that results from an injury to the perinatal brain. Individuals with CP present with well-known motor deficits such as muscle weakness, spasticity and altered motor control. In addition, they also present with sensory deficits, which can be attributed to both central and peripheral issues in the sensory system. Centrally, deficits in processing sensory information have been indicated through numerous neuroimaging studies, showing disrupted thalamocortical pathways and aberrant somatosensory cortical activation [1–4]. Peripherally, the sensory deficits present as greater sensory detection thresholds on clinical tests of lower extremity, such as aberrant two-point discrimination, light touch, hip and ankle joint position sense [5–8]. In summary, the sensory disorders can be attributed to impaired sensory feedback secondary to higher sensory detection thresholds or due to impaired feedforward mechanisms secondary to sensory processing deficits in CP. Individuals with CP compensate for sensory deficits, particularly in proprioception, by relying on vision over other senses for balance control [5, 9, 10]. Such excessive reliance on vision is associated with aberrant balance strategies in individuals with CP [11] and increased fall risk in other clinical populations [12–14].

Visual reliance for balance control has been established in standing and walking in individuals with CP. Visual manipulation of surrounding environment caused increased and variable body sway in individuals with CP compared to their typically developing peers [10]. Further, worsening of crouch stance was observed after removing visual input, thus indicating the dominant role visual input plays in control of standing balance in individuals with CP. With respect to walking, our recent work investigated how individuals with CP use visual input for walking balance control compared to their age-and sex-matched peers by subjecting them to visual sideways fall stimuli while walking in a virtual environment [15]. Our results showed that individuals with CP had a magnified and delayed response to visual perturbations, thus implying that they were more affected by changes in visual input and hence, relied more on vision for walking balance control.

The central nervous system adapts to changes in the environment by continuously adjusting the relative weight of different sensory sources such as vision, proprioception and vestibular system [16]. More reliable sensory inputs are weighed more strongly than less reliable inputs. The ability to upweight (i.e., increase the reliance on) the proprioceptive input as needed, especially in situations where one might receive insufficient or conflicting visual input, e.g., when moving from a well-lit to a dark room, is extremely important in maintaining upright balance. Children with typical development can reweight multisensory inputs from visual and proprioceptive sources from 4 to 6 years of age onwards [10, 17]. They were able to reduce their reliance on vision when receiving visual perturbations of increasing amplitude in standing. Individuals with CP also showed the ability to downweigh vision when large visual perturbations were provided. Thus, there is evidence of sensory reweighing in individuals with CP. Sensory reweighing has been used to reduce responses to visual perturbations by upweighing proprioceptive input in healthy young adults [18]. They reduced center of mass (CoM) displacement in response to translational perturbations after receiving proprioceptive augmentation through vibrotactile cues. It is not known if children with CP will similarly be able to upweight proprioceptive feedback and show reduced reliance on vision if they receive augmented proprioceptive feedback.

**Fig. 1:**
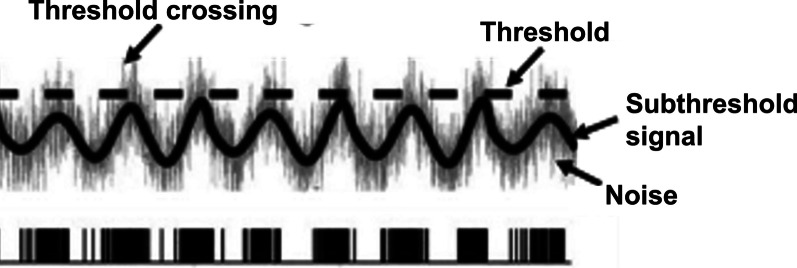
Top panel **A** depicts a subthreshold signal (thick black line) that crosses the threshold (dashed black line) after addition of noise (gray). Bottom panel **B** depicts the action potentials fired at threshold crossings, leading to spike trains. Adapted with permission from Moss et al. [19]

A novel and promising method to improve sensory feedback is application of stochastic resonance stimulation (SR). SR is a phenomenon where random, sub-sensory noise improves the ability of the non-linear systems to detect a signal. The SR phenomenon has been observed in a variety of biological systems, including visual, auditory, somatosensory and motor systems [19, 20]. The neurophysiological mechanism behind this phenomenon is that the subthreshold noise causes small changes in the transmembrane potentials of sensory receptors, making the sensory neuron more likely to fire an action potential in the presence of a weak stimulus (Fig. 1) [19]. Thus, in theory, application of SR would make a weak proprioceptive signal more likely to cross the sensory perception threshold and thus become more detectable [21, 22]. SR stimulation has improved standing balance in several clinical populations, such as patients with functional ankle instability (FAI) [23, 24], diabetic neuropathy, stroke, and in older adults [25, 26]. Specifically with respect to walking, older adults and frequent fallers have shown reduced variability in their spatiotemporal gait parameters after SR stimulation [27, 28]. In individuals with CP, SR stimulation significantly reduced postural sway in standing [29] and during regular, unperturbed walking [30]. However, the potential of SR stimulation during a more dynamic and functionally challenging task such as responding to visual perturbations is not yet explored. The typical response to a visual perturbation is to move the body’s center of mass (CoM) away from the direction of fall stimulus [31]. By upweighting proprioceptive input, SR may reduce the CoM response to visual stimulus and decrease the dependence on visual input for balance control, thus freeing visual information for high-level use such as navigation and obstacle avoidance.

The two biomechanical mechanisms typically used to modulate lateral CoM movement during walking are: (a) ankle roll, which involves using lateral ankle musculature to bring about inversion at the stance ankle and pull the body to one side, [32, 33], and (b) the foot placement, which involves stepping in the direction of a perceived fall to help accelerate the movement of the body away from the fall in subsequent steps [31, 34]. Prior work [15] showed that compared to typically developing peers, individuals with CP respond to visual perturbations with reduced ankle roll and increased foot placement change. In this study, we seek to investigate how these underlying biomechanical mechanisms are affected by potentially augmented proprioceptive input through SR.

The primary aim of this study is to investigate whether SR stimulation reduces the reliance on vision in individuals with CP compared to age- and sex-matched typically developing peers (TD) during visually perturbed walking. We hypothesize that SR stimulation will reduce the CoM responses to visual perturbations compared to the no SR stimulation condition, with greater reduction in the CP group compared to TD. Our secondary hypothesis is that SR stimulation would decrease the ankle roll and foot placement response, which would in turn be the mechanism for the hypothesized decrease in overall CoM response in the CP group.

## Methods

### Participants

We recruited 17 ambulatory individuals with spastic diplegic or hemiplegic CP through advertisements at local hospitals. Seventeen age-matched (± 6 months) and sex-matched typically developing individuals (TD) were recruited through advertisements and social media. Our CP group specifically included individuals with Gross Motor Function Classification (GMFCS levels I-II) to enable completion of the visual perturbation walking protocol without relying on a handrail for tactile cues. All participants were screened by a physical therapist for the inclusion and exclusion criteria listed in Table 1.

**Table 1:** Inclusion and exclusion criteria

Inclusion	Exclusion
Age 8–24 years Diagnosis of spastic diplegic or hemiplegic CP GMFCS classification level I or II (ability to walk independently with using any assistive device) Visual, perceptual, and cognitive/ communication skills to follow multiple step commands Seizure-free or well controlled seizures Ability to communicate pain or discomfort during testing procedures Parental/guardian consent and child assent/consent	Diagnosis of athetoid, ataxic or quadriplegic CP Significant scoliosis (scoliometer angle > 9°) History of selective dorsal root rhizotomy Botox injections in the lower limb within the past 6 months Severe spasticity of the lower extremity muscles (e.g. a score of 4 on the Modified Ashworth Scale) Severely limited range of motion/irreversible muscle contractures Lower extremity surgery or fractures in the year prior testing^*^ Joint instability or dislocation in the lower extremities^*^ Marked visual or hearing deficits^*^

*Denotes criteria applicable to TD group

To analyze the heterogenous group, we analyzed the data by considering the more affected and less affected sides separately. We determined the more affected side as the one with hemiplegia in individuals with hemiplegic CP, the one self-identified as the more affected side in individuals with diplegic CP and the non-dominant side in the TD group. The TD group self-determined their dominant side as their preferred lower limb of use during daily activities. The University of Delaware Institutional Review Board provided ethical oversight and approved the study protocol, which is registered at clinicaltrials.gov (NCT05233748). Informed parental consent and child assent were obtained.

### Instrumentation

Participants walked on a split-belt treadmill with the belts tied to operate synchronously (Bertec Inc., Columbus, Ohio, United States) in a virtual reality domed screen that completely covered their field of vision. The virtual scene comprised a 4-m wide, infinitely long corridor made of floating cubes and a checkered floor (Unity 3d, Unity Technologies, San Francisco, CA, United States). The perspective in the virtual world adapted in real time to the participant’s head movement by being linked to two infrared markers on the forehead. The treadmill was user self-paced through a custom labview program (National Instruments Inc., Austin, TX, United States) such that the speed of the treadmill adapted in real time to the participant’s self-selected walking speed.

Each visual perturbation or virtual “fall” began at the heelstrike of either foot. A stimulus consisted of rotating the virtual scene around an anterior–posterior axis of the treadmill with an angular acceleration of 45º/s^2^ for 600 ms. The scene remained tilted for 2000 ms and then reset to the horizontal over the next 1000 ms with a constant angular velocity. These perturbations mimic the optic flow of falling sideways around the stance foot and have been used extensively in our previous work [15, 31, 33, 35]. The perturbations were triggered at pseudo-randomly selected heel strikes of either foot, where each such trigger was followed by a 10–12 step washout period between the reset of the visual scene and the next trigger. To distinguish the response that was entirely due to visual perturbations from the regular body sway during unperturbed, steady-state walking, we alternated between triggers with an actual perturbation as described above and triggers with no perturbation, i.e., the participant continued walking in a regular, unperturbed manner.

We measured full body kinematics using a 13-camera motion capture system (Qualisys Inc., Gothenberg, Sweden). We used a full body Plug-in Gait marker set [36] with an additional marker on 5th metatarsal head bilaterally and six additional markers on the anterior thigh and shank. We recorded marker data at 200 Hz and ground reaction forces at moments at 1000 Hz. We low pass filtered the force plate data with 4th order Butterworth filter at a cut-off frequency of 20 Hz. We performed inverse kinematics for a 15-segment biomechanical model using OpenSim 4.0 [37]. We further processed the data to compute the below mentioned outcome measures (See section on Outcome Measures) using custom scripts in MATLAB.

### SR stimulation

**Fig. 2:**
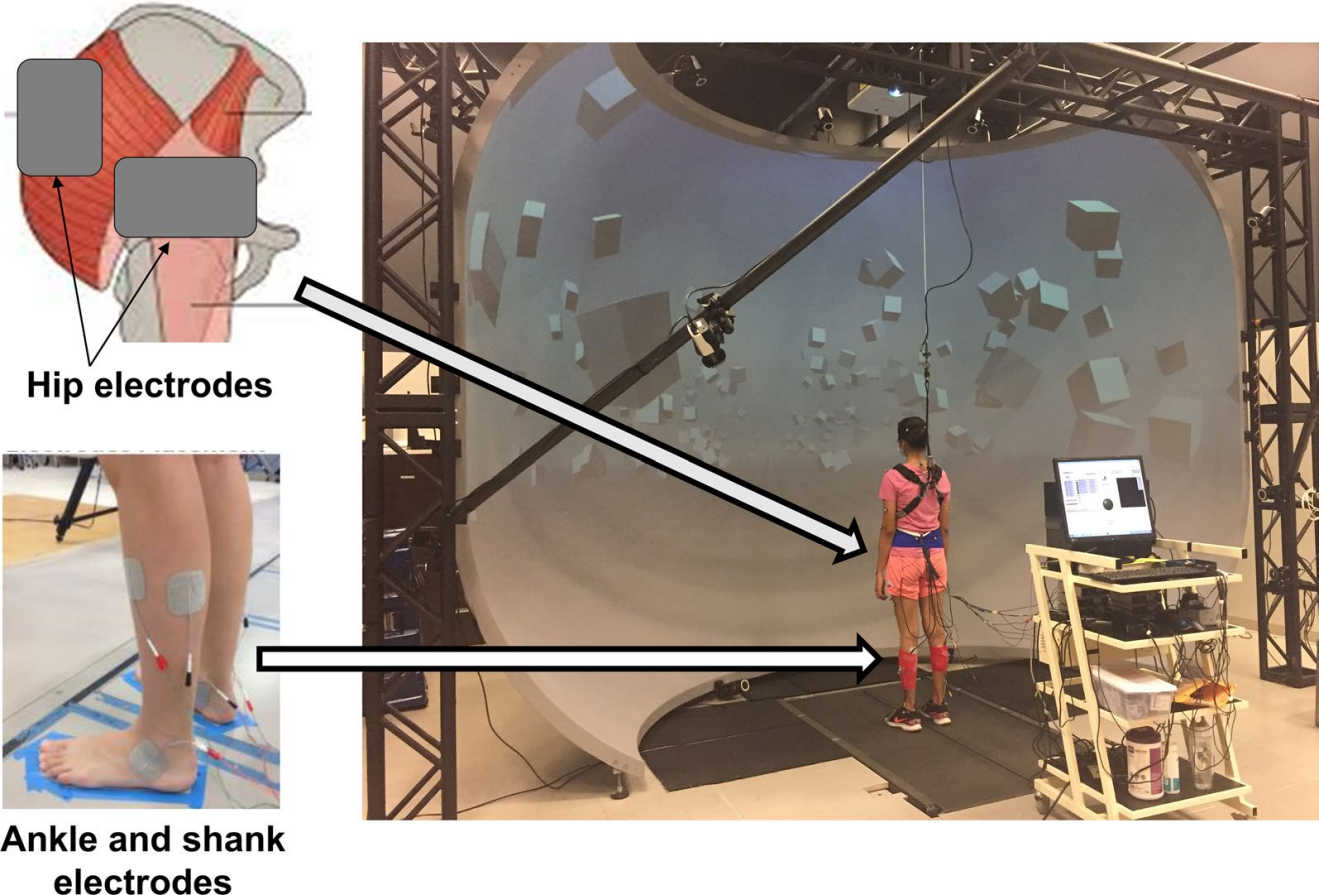
Experimental setup with the computer that generated the SR signal and six stimulators that delivered SR stimulation via surface electrodes at the hip, shank and ankle. The electrode leads were long enough to allow unencumbered walking at self-selected pace on the treadmill and were secured around the shank using a 3M Coban self-adhesive wrap. Figure reproduced from Sansare et al. [30]

A custom Labview program was used to generate the SR signal (Uniform White Noise) driving 6 stimulators (STMISOLA, Biopac Systems, Inc., Goleta, USA). SR intensity was defined as the amplitude of the interval of the uniform white noise. We placed self-adhesive electrodes over the ankle (anterior talofibular and deltoid ankle ligaments), shank (lateral soleus and peroneus longus, and tibialis anterior muscles) and at the hip (inferior and posterolateral, respectively, to the greater trochanter to stimulate the hip joint capsule and gluteus medius, and gluteus maximus). The set-up is shown in Fig. 2.

### Protocol

Participants were given at least two 2-min practice trials, one without any visual perturbations to get accustomed to the self-pacing treadmill and another 2-min trial with the visual perturbations to get comfortable with the virtual environment and the visual perturbation.

The experimental protocol included the following steps:

(1) Determine individual SR Sensory threshold: As a reference value for the range of possible intensities for SR stimulation, we determined each subject’s individual sensory detection thresholds at each stimulation site. A sensory threshold was defined as the minimum level of stimulation required for an individual to detect a mild tingling sensation. Participants walked on the treadmill at a self-selected fixed speed while the stimulation intensity was increased in increments of 0.1 mA until the participant reported feeling the sensation. To verify this threshold, we decreased the intensity until the participant could no longer feel the stimulation. We repeated this procedure thrice and the sensory threshold for that site was the lowest stimulation intensity over the three repetitions.

(2) SR Optimal Intensity: For stochastic resonance to boost the detection of proprioceptive signals, a specific optimal level of SR stimulation intensity is required. To find the optimal intensity for each participant, we tested balance performance at 25%, 50%, 75%, and 90% of the participant-specific sensory threshold derived in the previous step. Each participant walked for 2 min at each intensity on the treadmill with rest breaks in between each trial. The SR intensities were presented in randomized order via a computerized protocol. We assessed balance performance for each intensity by calculating the minimum lateral margin of stability (MOS), a measure previously used for characterizing balance control during walking in children with and without CP [38]. A larger MOS implies a larger impulse is needed to become unstable, and in turn implies higher stability. The SR intensity that is the most protective against a lateral fall i.e., greatest increase in the MOS, among the four SR intensities was defined as the individual’s optimal intensity (SRopt) level and used for subsequent testing. Because the SR intensities are sub-threshold, i.e., below 100% of sensory threshold, the participants did not perceive the stimulation and were blinded to the different SR intensities at all times.

(3) Visual Perturbation Protocol: To investigate the effect of SR stimulation on balance, we used SR stimulation at optimal intensity (SRopt) and no stimulation (no SR) as a control condition. Participants completed three trials each of 2-min in length under each of the two (SRopt and noSR) conditions while receiving visual perturbations as described above. The SRopt and noSR conditions were presented in randomized order and the subjects were blinded to either condition.

### Outcome measures

Our primary outcome measure to quantify the effect of SR on response to visual perturbations was the area under the curve of the M-L CoM excursion (AUC M-L CoM excursion). The CoM excursion was defined as the difference between the average CoM for the perturbed steps from control (no perturbation) steps for each participant, which was then integrated over the eight steps following the heel strike that triggered a stimulus. To quantify the extent of the CoM excursion, we determined the peak of the CoM excursion over the same period (Peak M-L CoM excursion). To quantify the timing of the response, we determined the time between the onset of the perturbation and the peak CoM excursion (Peak Time). Secondary outcome measures are ankle roll and foot placement responses. We quantified the ankle roll by calculating the subtalar angle at the stance leg, integrated over the first single stance period following the perturbation (AUC subtalar angle). For the foot placement response, we used the medial–lateral location of the leading foot relative to the trailing foot at heel strike. We first fit a linear model to predict the foot placement from the CoM state at midstance during regular walking [39] and used the difference from this regression line at each step as the outcome measure for foot placement. We then calculated the average foot placement response over the first three post-perturbation steps as a measure of the overall foot placement response following a visual perturbation. These outcome measures have been previously used to assess the response to visual perturbations in individuals with CP [15] and in neurotypical healthy adults [31, 35].

### Statistical analysis

#### Statistical power

Thirty-four participants divided equally over two groups (17 CP, 17 TD) were recruited. The sample size was determined through an a priori power analysis using a significance α = 0.05, power of 0.80 to detect a medium effect size (f = 0.25) in G Power (Version 3.1.9.4).

#### Analysis

We performed two-way mixed ANOVAs, with group (CP, TD) as the between subject factor and condition (noSR, SR) as the within-subject factor. We analyzed the more affected and less affected side separately. Pairwise post hoc comparisons were performed using Bonferroni tests. We assessed assumptions of homoscedasticity and normality, respectively, by Levene’s and Shapiro–Wilk tests in addition to visual examination. Between-group differences for baseline characteristics such as age and body mass index (BMI) were assessed using paired samples t-test.

## Results

While participants in both groups were challenged with the visual perturbations, none of the participants stepped off the treadmill or fell over in the safety harness. Out of 34 participants, 31 participants responded to the visual perturbations by moving their CoM away from the direction of virtual fall, which is as expected. However, two participants from the CP group responded by moving their CoM towards the direction of the fall while the third participant responded by moving the CoM vertically lower to the ground rather than moving it in a mediolateral plane. Because these responses are not representative of a group-wide behavior, we chose to exclude these three participants and their corresponding TD controls from the statistical analysis. The demographic characteristics of both groups are reported in Table 2.

**Table 2:** Mean, standard deviation (SD) and p values for the difference between the CP ad TD groups for demographic and spatiotemporal gait variables

	CP (n = 14)	TD (n = 14)	p value
	Mean ± SD	Mean ± SD	
Age (years)	16.3 ± 4.3	16.1 ± 4.2	0.27
Height (meters)	1.61 ± 4.3	1.65 ± 0.13	0.16
Weight (kg)	50.3 ± 12.2	65.4 ± 17.7	0.003
BMI (kg/m^2^)	19.0 ± 3.1	23.6 ± 4.7	0.011
Cadence (steps/min)	113 ± 12	106 ± 7	0.07
Normalized Velocity (sec)	1.306 ± 0.263	1.167 ± 0.531	0.419
Step Width (m)	0.143 ± 0.063	0.095 ± 0.041	0.012
Normalized Step Length: More affected side	0.677 ± 0.105	0.743 ± 0.087	0.105
Normalized Step Length: Less affected side	0.675 ± 0.105	0.745 ± 0.082	0.082
Step Time: More affected side (sec)	0.534 ± 0.071	0.564 ± 0.040	0.137
Step Time: Less affected side (sec)	0.551 ± 0.067	0.574 ± 0.041	0.243
% Double Support Time	35.08 ± 3.50	37.85 ± 2.51	0.005

Velocity and step length are normalized to leg length (meters)

The descriptive statistics for all outcome measures are presented in Additional file 1: Table S1. The full details of the statistical analysis, including p-values, degrees of freedom and effect sizes are provided in Table 3.

**Table 3:** Degrees of freedom (df), F statistic, p value and effect sizes (partial eta square)

	Outcome measure	Effect	df	F statistic	p value	Partial eta squared
Affected Side	AUC M-L COM excursion (meters·sec)	Group	[1,26]	0.072	0.791	0.003
		Condition	[1,26]	9.464	0.005	0.267
		Group*condition	[1,26]	9.491	0.005	0.267
	Peak M-L COM excursion (meters)	Group	[1,26]	0.427	0.519	0.016
		Condition	[1,26]	9.237	0.005	0.262
		Group*condition	[1,26]	7.812	0.010	0.231
	Peak Time (seconds)	GROUP	[1,26]	0.086	0.772	0.003
		Condition	[1,26]	3.580	0.070	0.121
		Group*condition	[1,26]	1.608	0.216	0.058
	Subtalar angle (degrees·sec)	Group	[1,26]	0.231	0.635	0.009
		Condition	[1,26]	1.042	0.317	0.039
		Group*condition	[1,26]	0.304	0.586	0.012
	Foot Placement-averaged over 3steps (meters)	Group	[1,26]	4.802	0.038	0.156
		Condition	[1,26]	0.001	0.974	0.001
		Group*condition	[1,26]	0.053	0.820	0.002
Less Affected Side	AUC M-L COM excursion (meters·sec)	Group	[1,26]	14.051	< 0.001	0.351
		Condition	[1,26]	2.722	0.111	0.095
		Group*condition	[1,26]	0.903	0.351	0.034
	Peak M-L COM excursion (meters)	Group	[1,26]	14.165	< 0.001	0.353
		Condition	[1,26]	1.618	0.215	0.059
		Group*condition	[1,26]	3.566	0.070	0.121
	Peak Time (seconds)	Group	[1,26]	0.004	0.947	0.001
		Condition	[1,26]	0.218	0.645	0.008
		Group*condition	[1,26]	2.014	0.168	0.072
	Subtalar angle (degrees·sec)	Group	[1,26]	0.455	0.506	0.017
		Condition	[1,26]	1.227	0.278	0.045
		Group*condition	[1,26]	4.526	0.043	0.148
	Foot Placement-averaged over 3steps (meters)	Group	[1,26]	0.879	0.357	0.033
		Condition	[1,26]	0.039	0.844	0.002
		Group*condition	[1,26]	2.403	0.133	0.085

Partial eta square of 0.01 indicates a small effect, 0.06 indicates a medium effect, 0.14 indicates a large effect

### Center of mass response

#### AUC ML CoM excursion

**Fig. 3:**
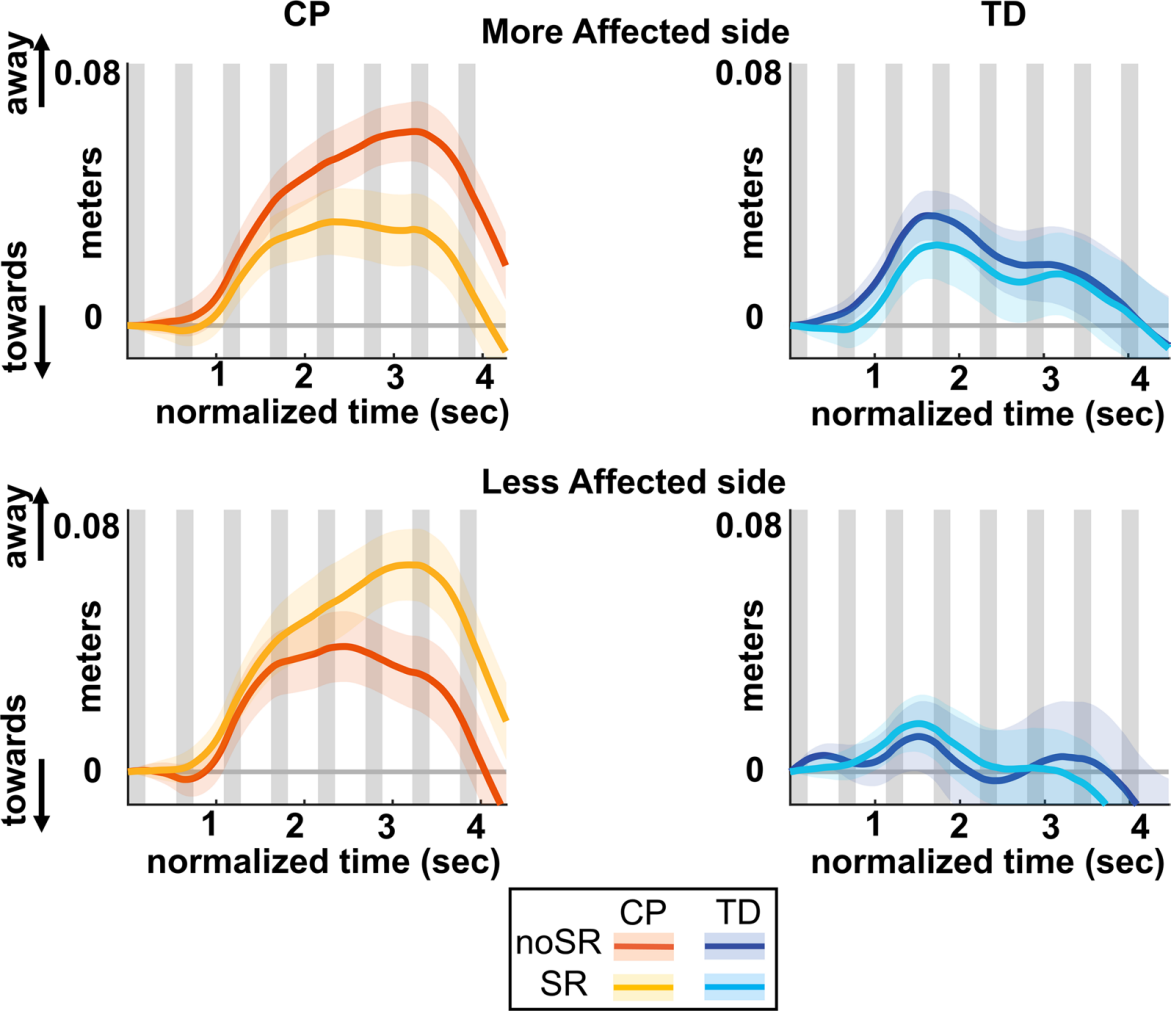
Group average trajectories for medio-lateral center of mass excursion in response to visual fall stimuli for both noSR and SR conditions in CP (orange: noSR, yellow: SR) and TD (dark blue: noSR, light blue: SR) on the more affected (left panel) and less affected (right panel) side. Thick gray line along zero at X-axis indicates the mean of control (no fall stimulus) steps, which is subtracted from stimulus data. Shaded areas around each trajectory represent 95% confidence interval. X-axis shows 8 steps, time-normalized to 100 timepoints per steps, with double-stance (gray shading) and single-stance (no shading)

**Fig. 4:**
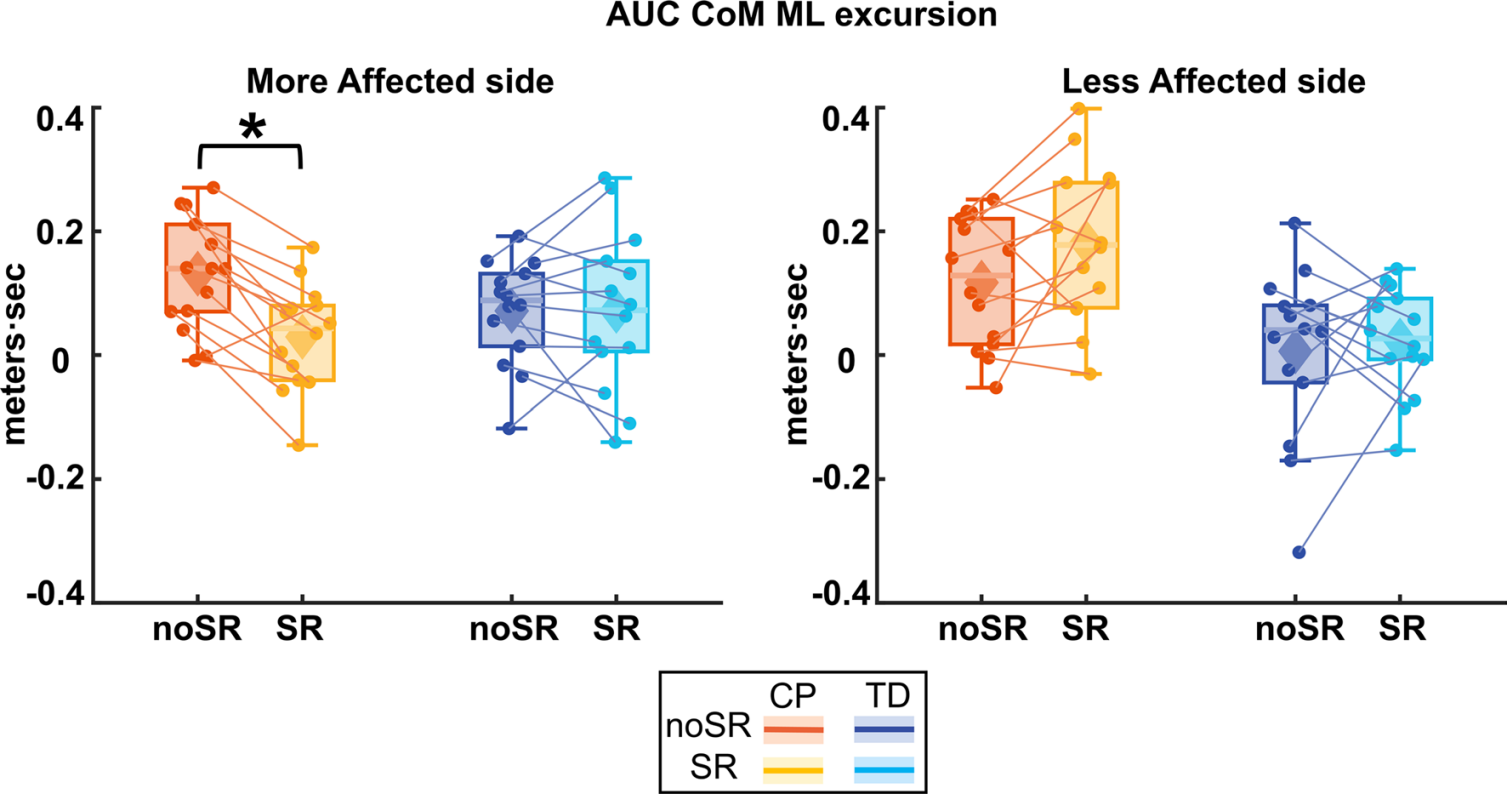
Box and whisker plots, with scattered dots indicating each participant for area under the curve of mediolateral center of mass excursion (AUC CoM M-L excursion) for both noSR and SR conditions in CP (orange: noSR, yellow: SR) and TD (dark blue: noSR, light blue: SR) on the more affected (left panel) and less affected (right panel) side. Asterisk indicates p < 0.05

Figure 3 shows the average ML CoM excursion over eight post-perturbation steps. Figure 4 shows the box and whisker plots for AUC CoM ML excursion. For the more affected side, there was a significant group by condition interaction for AUC ML CoM excursion (p = 0.005), which indicates that the response to SR differed depending on which group the participants belonged to. Pairwise post hoc comparisons using Bonferroni corrections showed that in the CP group, the average AUC ML CoM excursion reduced significantly with SR compared to the noSR condition (p < 0.001), whereas the TD group did not show a significant change with SR compared to noSR (p = 0.998). For the less affected side, while both groups seemed to show an increase in the AUC ML CoM excursion, there was no significant effect for condition (p = 0.111) nor a group by condition interaction (p = 0.351).

#### Peak CoM excursion

**Fig. 5:**
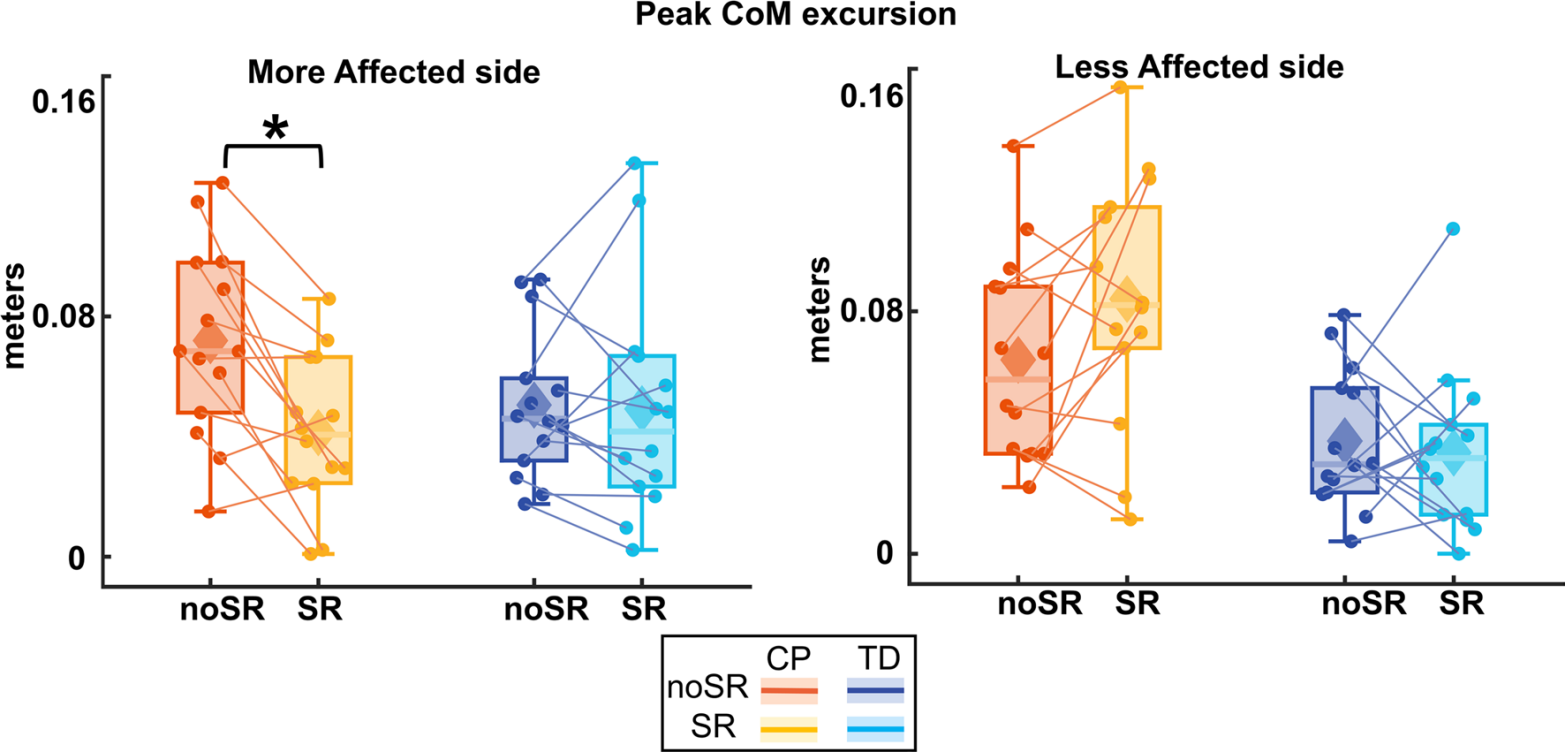
Box and whisker plots, with scattered dots indicating each participant for peak center of mass mediolateral excursion (Peak CoM ML excursion) for both noSR and SR conditions in CP (orange: noSR, yellow: SR) and TD (dark blue: noSR, light blue: SR) on the more affected (left panel) and less affected (right panel) side. Asterisk indicates p < 0.05

Figure 5 shows the box and whisker plots for peak CoM excursion. For the more affected side, there was a significant group by condition interaction for Peak CoM excursion (p = 0.010). Pairwise post hoc comparisons using Bonferroni corrections showed that in the CP group, the average peak excursion reduced significantly with SR compared to the noSR condition (p < 0.001), whereas the TD group did not show a significant change with SR compared to noSR (p = 0.864). For the less affected side, there was no statistically significant effect for condition (p = 0.215) nor a group by condition interaction (p = 0.070).

#### Peak time

**Fig. 6:**
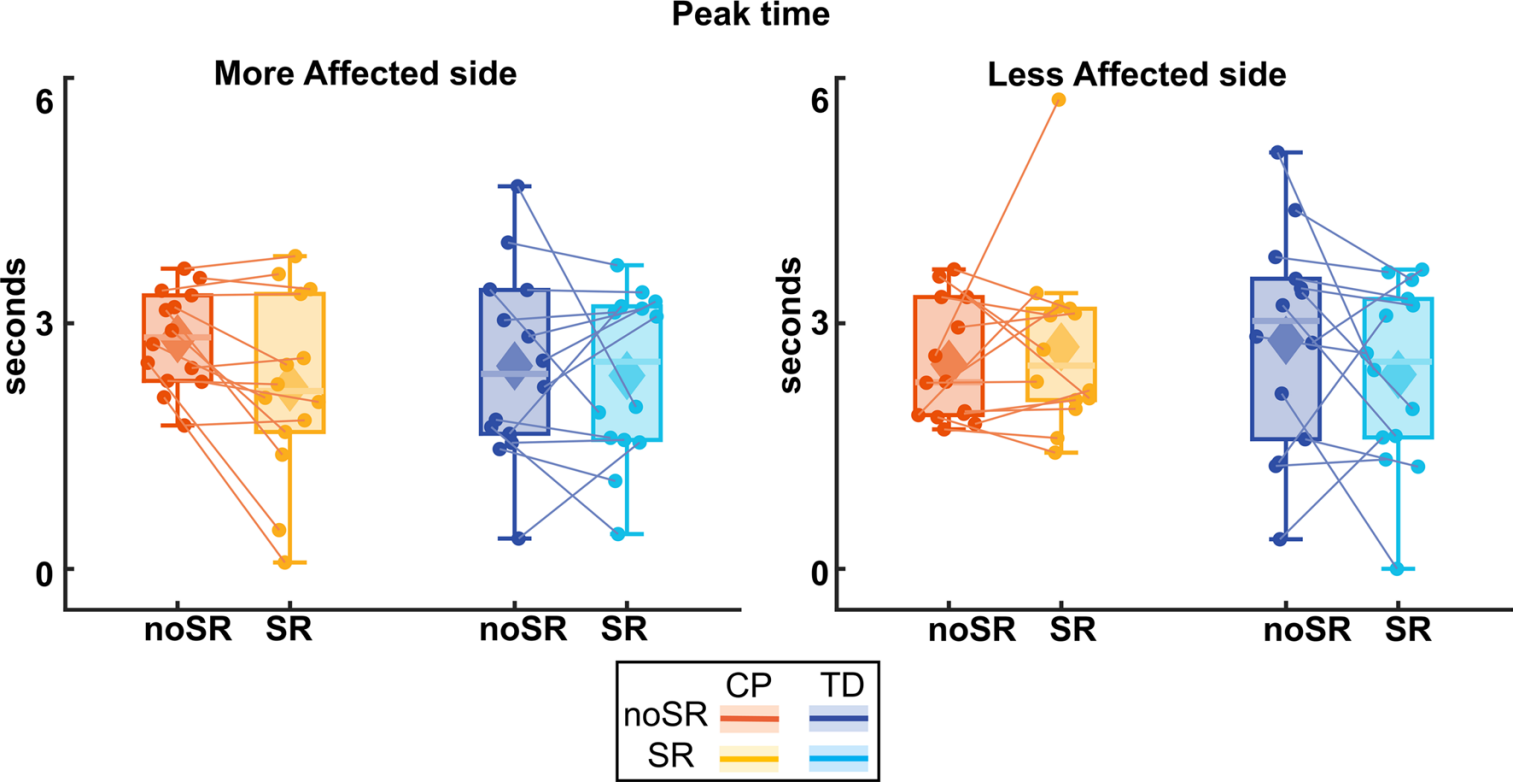
Box and whisker plots, with scattered dots indicating each participant for peak time of center of mass mediolateral excursion (Peak Time) for both noSR and SR conditions in CP (orange: noSR, yellow: SR) and TD (dark blue: noSR, light blue: SR) on the more affected (left panel) and less affected (right panel) side

Figure 6 shows the box and whisker plots for peak time. For the more affected side, while the CP group did reduce their peak time by about 600 ms with SR compared to noSR, there was no statistically significant effect for condition (p = 0.070) nor a group by condition interaction (p = 0.216). For the less affected side, CP group increased their peak time with SR compared to noSR, however, there was no significant effect for condition (p = 0.645) nor a group by condition interaction (p = 0.168).

### Ankle roll response

**Fig. 7:**
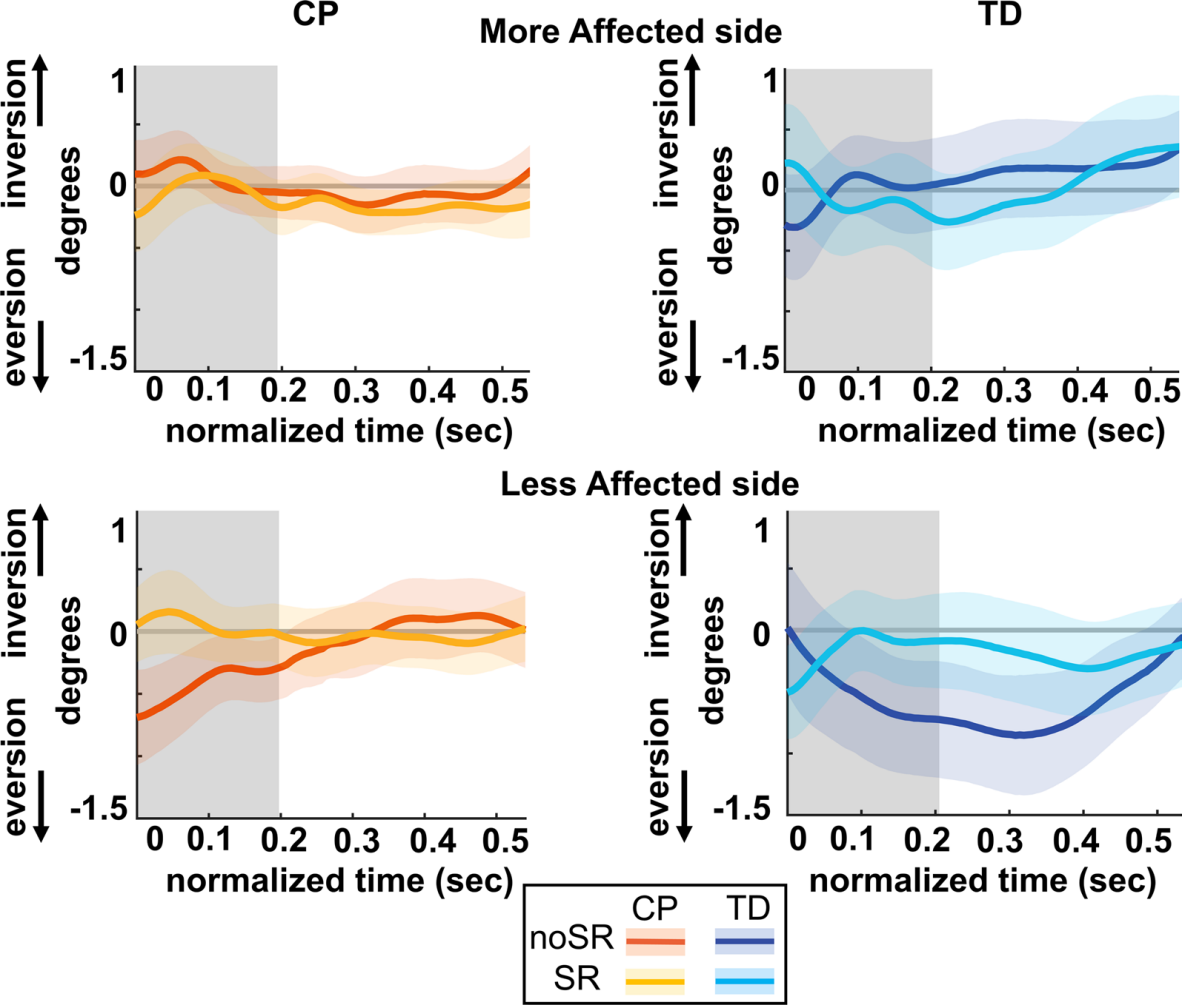
Group average trajectories for subtalar angle during the first post-stimulus step following a visual fall perturbation for both noSR and SR conditions in CP (orange: noSR, yellow: SR) and TD (dark blue: noSR, light blue: SR) on the more affected (left panel) and less affected (right panel) side. Thick gray line along zero at X-axis indicates the mean of control (no fall stimulus) steps, which is subtracted from stimulus data. Shaded areas around each trajectory represent 95% confidence interval. X-axis shows 8 steps, time-normalized to 100 timepoints per steps, with double-stance (gray shading) and single-stance (no-shading). Positive and negative Y-axis indicates inversion and eversion, respectively

**Fig. 8:**
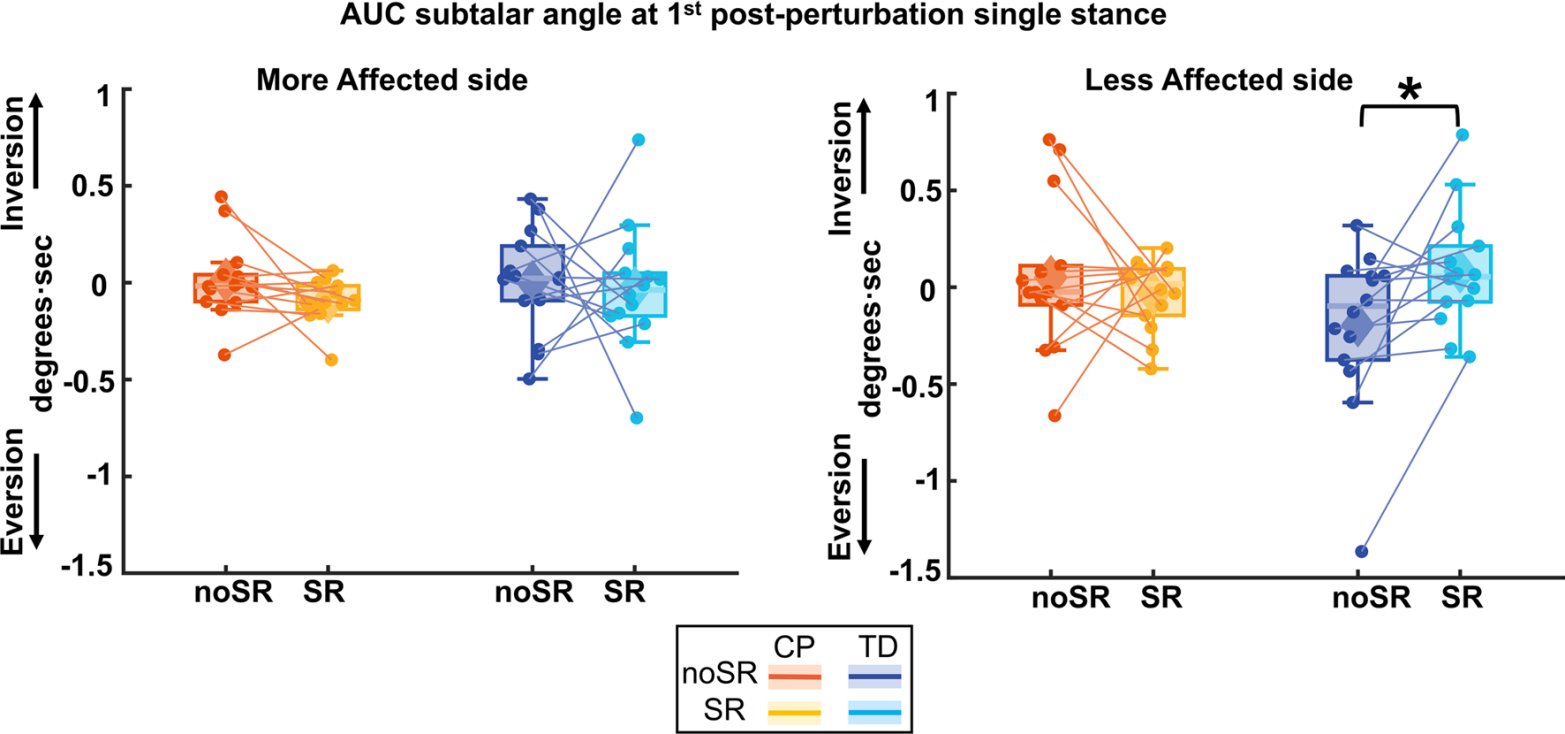
Box and whisker plots, with scattered dots indicating each participant, for AUC subtalar angle for both noSR and SR conditions in CP (orange: noSR, yellow: SR) and TD (dark blue: noSR, light blue: SR) on the more affected (left panel) and less affected (right panel) side. Asterisk indicates p < 0.05

Figure 7 shows the average subtalar angle over the first post-perturbation step. Figure 8 shows the box and whisker plots for subtalar angle. For the affected side, both groups showed a reduction in the AUC subtalar angle with SR compared to noSR, indicating a reduced ankle inversion or a reduced ankle roll response. However, there was no significant condition effect (p = 0.317) nor a group by condition interaction (p = 0.586). For the less affected side, there was a significant group by condition interaction (p = 0.043). Pairwise post hoc comparisons using Bonferroni corrections showed that in the TD group, the average AUC subtalar angle increased significantly with SR compared to the noSR condition (p = 0.031), however, this change was minimal and could be potentially clinically irrelevant whereas the CP group did not show a significant change with SR compared to noSR (p = 0.477).

### Foot placement response

**Fig. 9:**
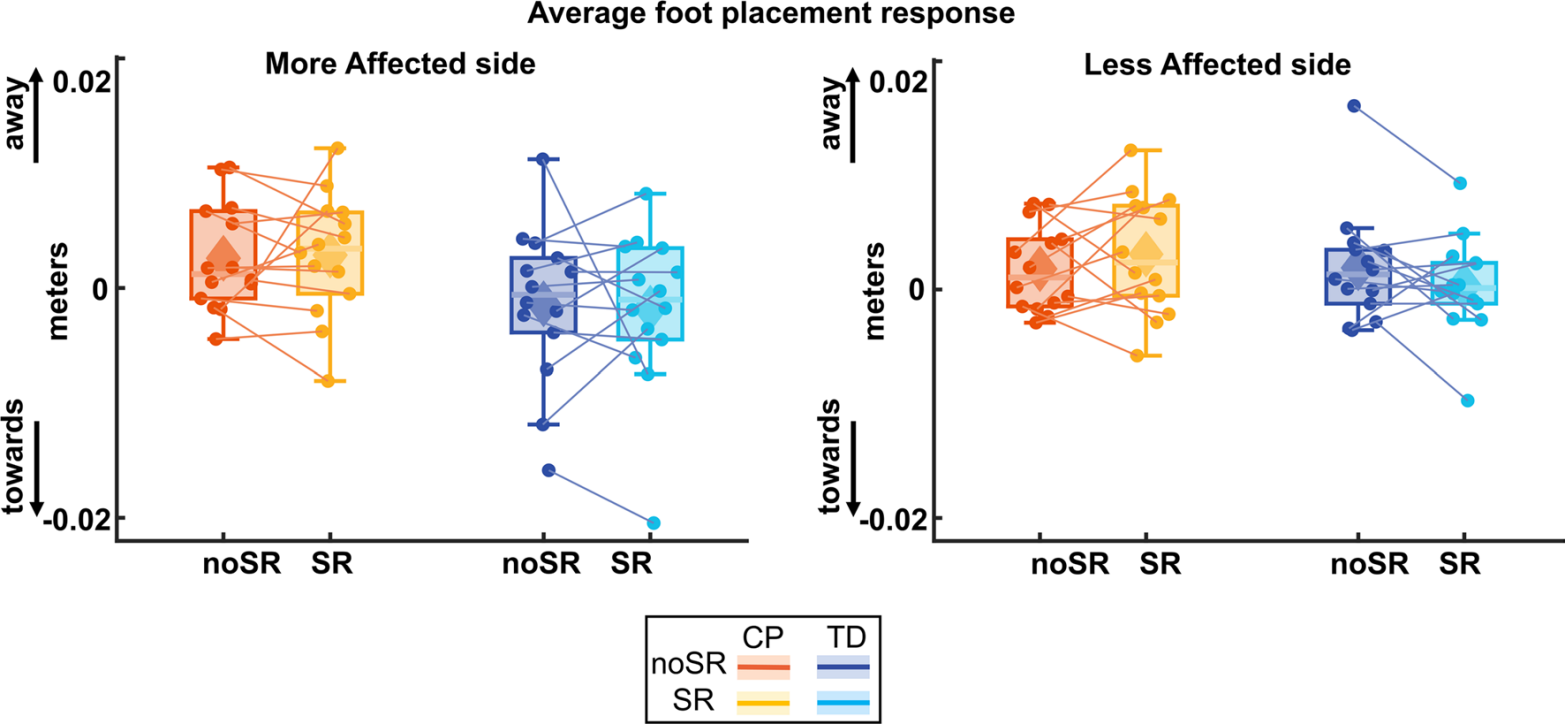
Box and whisker plots, with scattered dots indicating each participant, for average foot placement response over first three post-perturbation steps for both noSR and SR conditions in CP (orange: noSR, yellow: SR) and TD (dark blue: noSR, light blue: SR) on the more affected (left panel) and less affected (right panel) side

Figure 9 shows the average foot placement over the first three post-perturbation steps. For the more affected side, there was no significant effect for condition (p = 0.974) nor a group by condition interaction (p = 0.820). Similarly, for the less affected side, there was no significant effect for condition nor a group by condition interaction (p = 0.844) nor a significant effect for condition (p = 0.133).

## Discussion

In this study, we investigated how the response of individuals with and without CP to visual perturbations changed with application of a sensory-centric therapy such as SR stimulation. Our hypothesis was partially validated, in that the CP group showed a reduced response to the visual perturbations with SR compared to sham stimulation whereas the delay in generating the response did not improve with SR. The TD group did not show any change with or without SR in the CoM response to visual perturbations. This improvement in the response to visual perturbations was only seen on the more affected side, whereas there was no significant change with or without SR for either group on the less affected side. However, contrary to our hypothesis, the large reduction in the CoM response was not mirrored in the two mechanisms typically responsible for the CoM response, ankle roll and foot placement response. Thus, while our study provides compelling evidence that SR stimulation helped individuals with CP to reduce their response to visual fall stimuli, implying that they were less affected by visual perturbations with SR, the specific mechanisms responsible for the CoM response remain unclear.

Prior work on the response of individuals with CP to visual perturbations compared to their TD peers has shown that they have a magnified and delayed CoM response. With SR stimulation, the CP group reduced their overall CoM response to visual perturbations, as indicated by a reduced AUC for CoM excursion, compared to the perturbation trials without SR on the more affected side. A magnified CoM response during walking and standing in individuals with CP [10, 11, 15] and during walking in older adults [40] suggests increased reliance on vision over other senses, particularly impaired proprioception, for balance control. Thus, a reduction in the CoM response to visual perturbation suggests a reduced reliance on vision and a potential upweighting of proprioception via SR stimulation. Lastly, the peak CoM excursion, which indicates the magnitude of how far the CoM travelled after a visual perturbation, also showed similar findings as the AUC CoM excursion. The peak time, which is indicative of the delay in generating the peak CoM response, also reduced by ~ 600 ms with SR. While this difference was not statistically significant, given that the average peak time for the TD group was around 2 s, a reduction of this magnitude is critical from a neuromotor control standpoint.

While we found statistically significant improvements in CoM response with SR stimulation on the more affected side, the opposite results, i.e., an increase in the CoM response (though not statistically significant), was observed with SR on the less affected side in both groups. Why is the response so different on the affected vs. the less affected side? One possible explanation is that we selected the optimal SR intensity as the one that resulted in the greatest increase in MoS, i.e. the intensity that best increased the stability, on the more affected side. Although this intensity was selected to optimize balance control on the more affected side, it was applied evenly to perturbations triggered by heelstrikes of either side as part of the randomized protocol. We made these decisions in the protocol design for two reasons. First, based on our prior work that did not show any side-specific differences in the responses to visual perturbations, we did not expect the more and less affected side to behave differently. Second, it was not feasible for the studied pediatric population to repeat the measurements with an intensity selected for optimal effect on the less affected side because it would have made the protocol longer and tiresome, particularly for the younger participants in our cohort. Thus, while this optimal intensity may have been tailored to the more affected side, which is where we expected the most deficits to be present, it may have resulted in too little or too much stimulation for the less affected side. Two studies have specifically investigated the use of different ranges of subthreshold and suprathreshold intensities i.e., intensities below and above the sensory threshold, respectively, for SR stimulation. Severini et al. [41] showed that subthreshold SR intensities, such as 70% or 90% of the sensory threshold, improved the postural sway while suprathreshold SR, such as 100% and 130% of the sensory threshold, led to increased postural sway. Cordo et al. [22] showed that an inverted U-shaped phenomenon exists with respect to SR, where intensities above and below a certain subject-specific optimal level are not successful in improving the detection of the sensory signal. Both these studies point towards the ineffectiveness of SR if the intensity is not carefully selected to be optimal for the specific task. Thus, overdosing or underdosing on the less affected side may be the reason for the observed worsening of response to visual stimulations with SR stimulation.

It is interesting to note that even in the TD group, where we expected no interlimb differences between the more and less affected side, the more affected side had a greater response to visual perturbation compared to the less affected side. The more and less affected side in TD were determined based on their dominant side. Thus, the differences in TD children between the more and less affected side may be due to differences in the sensory abilities between the dominant and less dominant side. However, there is currently no consensus on the effect of lower limb dominance on postural control. Firstly, lower limb dominance is determined in several ways across multiple studies, such as self-selected preferred leg, through a footedness questionnaire, the leg chosen for kicking or a task specific activity such as jumping or stepping up. Secondly, the results of these studies are mixed, with some studies reporting no effect of lower limb dominance [42–47] while others show that lower limb dominance influences postural control in healthy adults [48–51]. Hence, future work can investigate whether differences in the sensory thresholds, and processing and integration of sensory input between the dominant and non-dominant lower limb contribute to differences in balance responses to visual perturbations between the more versus less affected side in typically developing children.

A change in the CoM response is expected to be accompanied by a corresponding change in the ankle roll and foot placement response, since these are the balance mechanisms driving the whole-body movement by modulating the force against the ground. Our prior work on responses to visual perturbations has shown that individuals with CP have reduced ankle roll and magnified foot placement response. With SR stimulation, we had expected a reduction in either or both the mechanisms. While the ankle roll response was reduced slightly in both groups, its magnitude was too small to drive such a large corresponding decrease in CoM response. Surprisingly, there was no change in the foot placement mechanism, which is generally considered to be the most effective means of generating a reduction in the CoM response of this magnitude [34]. It is possible that when we removed three subjects from the CP group during the statistical analysis, the reduced sample size may have increased the possibility of a false negative or a type II error. However, given the small effect sizes for the ankle roll and even smaller effect size for foot placement on more affected side, which is the side where the reduced CoM response occurred, it is unlikely that three additional participants in each group would have caused a significant change in the results. Another explanation for the mechanism behind the reduced CoM response on the more affected side is that there is a different balance mechanism, which is influenced by SR stimulation in individuals with CP that in turn drives their CoM response. Some examples of potential balance mechanisms influenced by SR that may be generating the observed decrease in CoM response could be the hip strategy i.e., the use of hip musculature to generate torques to pull on the trunk [52], the push-off strategy i.e., modulating the plantarflexion angle of the trailing leg ankle to accelerate the CoM towards the leading leg [53], a reduced vertical CoM excursion [54], use of arm swing for more effective recovery following perturbations [55], or a combination of two or more of these mechanisms. But given the heterogeneity seen in the clinical presentation and in the gait abnormalities in this population, an exploration of alternative balance mechanisms responsible for driving the CoM response is beyond the scope of this paper.

While the current experimental set-up for SR used in this study is several steps removed from being used in a clinical setting due to the long setup time and tedious experimental protocol, our results provide proof-of-concept that a sensory-based treatment approach can reduce visual reliance for walking balance control. These results will add to the current motor-centric treatments, thus providing a more comprehensive approach to balance rehabilitation. Improved balance will in turn lower incidence of falls and fall-related sequelae, and improve quality of life in individuals with CP.

### Limitations

Our study has several limitations to consider while interpreting our results. First, our study investigated only the immediate effects of SR application on the response to visual perturbations. While our results demonstrate the potential of SR in improving walking balance in an acute pre- versus post intervention design, we do not know (1) how long these improvements in balance last for i.e. we explicitly test SR against noSR in a design that temporally interlaces both with each other and if there was a carry-over effect, it would reduce the effect by SR trials carrying over into noSR trials directly after, and (2) whether the improvements can be retained with training program, both of which are important considerations for translating the obtained results in actual patient care. Second, sensory reweighing for balance control also involves a third sensory mode, the vestibular system, in addition to vision and proprioception. To focus on the interplay between two sensory systems our research question probed vision and proprioception, with the goal of allowing participants with CP to reduce their over-reliance on vision by improving information from proprioception. We did not actively manipulate the vestibular system through perturbations or stimulation in this protocol. We also screened out individuals with known history of any vestibular disorders and performed vestibular tests prior to beginning the study protocol to rule out any influence of impaired vestibular system on our results. Our results do not shed light on the role of vestibular contributions to balance control in CP and whether similar upweighting of proprioception would be observed if the vestibular system were experimentally perturbed. Third, we did not add any SR stimulation at the trunk. SR stimulation to improve postural sway and balance control has been typically applied at the ankle and foot, and leg muscles, such as tibialis anterior and gastrocnemius [24, 29, 56, 57]. In quiet standing, movement around the ankle joint has the largest effect on body sway [58], so improving proprioceptive information is most beneficial at the muscles and ligaments surrounding this joint. For mediolateral control of walking balance, the hip and trunk contribute substantially [33, 52, 59]. The hip joint is critical for lateral balance and has to transition from almost free movement during swing to bearing and stabilizing the majority of the body weight during stance. Experimentally altering proprioceptive signals at the stance leg hip joint using vibration leads to changes in foot placement for balance control [60]. While we accounted for the roles the ankle and hip play during walking by applying SR stimulation at the ankle, shank and hip joints, we did not add SR stimulation to the trunk. Future work that includes trunk stimulation in addition to hip, leg and ankle may produce larger improvements in balance responses to visual perturbations.

## Conclusion

Overall, our findings indicate that a sensory-centric therapeutic intervention, such as SR stimulation, resulted in reduced responses to visual perturbations in individual with CP compared to their age-and sex-matched peers. We propose that SR may have led to upweighting of proprioceptive input and downweighing of visual input, leading to a reduced reliance on vision for walking balance control. While SR has shown to be potentially effective in improving standing balance previously, these findings highlight the potential of SR in altering the integration and relative contributions of sensory input to actively control balance during walking. However, our current results do not pinpoint the exact balance mechanism that drive the observed improvements in the whole-body response and exploration of alternative balance mechanisms in a clinical population such as CP may be a topic for future research.

### Supplementary Information

**Additional file 1: Table S1.** Mean and 95% confidence interval from estimated marginal means for CP and TD groups on the more affected and less affected side under noSR and SR conditions.

## Data Availability

The data associated with this analysis are available from the corresponding author upon request.
