# Supplementary material for: Immediate application of low-intensity electrical noise reduced responses to visual perturbations during walking in individuals with cerebral palsy
Source: J Neuroeng Rehabil. 2024 Jan 28;21:14. doi: 10.1186/s12984-023-01299-1 (PMC10822182; doi:10.1186/s12984-023-01299-1)
Supplement: Supplementary file 1 — Additional file 1: Table S1. Mean and 95% confidence interval from estimated marginal means for CP and TD groups on the more affected and less affected side under noSR and SR conditions. [file 12984_2023_1299_MOESM1_ESM.docx]

Table S1: Mean and 95% confidence interval from estimated marginal means for CP and TD groups on the more affected and less affected side under noSR and SR conditions.
